# A magnetic sensor using a 2D van der Waals ferromagnetic material

**DOI:** 10.1038/s41598-020-61798-2

**Published:** 2020-03-16

**Authors:** Valery Ortiz Jimenez, Vijaysankar Kalappattil, Tatiana Eggers, Manuel Bonilla, Sadhu Kolekar, Pham Thanh Huy, Matthias Batzill, Manh-Huong Phan

**Affiliations:** 1 Department of Physics, University of South Florida, Tampa, FL 33620 USA; 2 Phenikaa Institute for Advanced Study, Phenikaa University, Yen Nghia, Ha-Dong District, Hanoi, 1000 Viet Nam

**Keywords:** Sensors and biosensors, Magnetic properties and materials

## Abstract

Two-dimensional (2D) van der Waals ferromagnetic materials are emerging as promising candidates for applications in ultra-compact spintronic nanodevices, nanosensors, and information storage. Our recent discovery of the strong room temperature ferromagnetism in single layers of VSe_2_ grown on graphite or MoS_2_ substrate has opened new opportunities to explore these ultrathin magnets for such applications. In this paper, we present a new type of magnetic sensor that utilizes the single layer VSe_2_ film as a highly sensitive magnetic core. The sensor relies in changes in resonance frequency of the LC circuit composed of a soft ferromagnetic microwire coil that contains the ferromagnetic VSe_2_ film subject to applied DC magnetic fields. We define sensitivity as the slope of the characteristic curve of our sensor, *df*_0_/*dH*, where *f*_0_ is the resonance frequency and *H* is the external magnetic field. The sensitivity of the sensor reaches a large value of 16 × 10^6^ Hz/Oe, making it a potential candidate for a wide range of magnetic sensing applications.

## Introduction

Since intrinsic long-range ferromagnetic order was realized in 2017 with bulk exfoliated monolayers of Cr_2_Ge_2_Te_6_ and CrI_3_^1,2^, the potential of two-dimensional (2D) van der Waals magnets has excited the scientific community^3–11^. While these 2D magnets have demonstrated their usefulness in magnetoelectric devices, their technological applications are restricted to low temperatures (<100 K)^6–10^. In contrast, recent discoveries of the room temperature (RT) ferromagnetism in transition metal dichalcogenide (TMD) monolayers of VSe_2_ and MnSe_2_ grown by van der Waals epitaxy on various substrates (graphite, MoS_2_, GaSe) may enable applications at ambient temperature^3,4,10^. Due to the nature of atomically thin materials, their physical properties are sensitive to external stimuli. In this regard, it would be very interesting to exploit the potential of 2D magnets for magnetic sensing applications. In this paper, we present a new type of magnetic sensor that integrates a single layer VSe_2_ film within a Co-rich microwire-based coil.

Induction coil sensors have been widely used due to the simplicity of their construction and a well known transfer function^12^. It is established that the sensivity of an induction coil is limited by the number of windings; the greater the number of turns the higher the sensitivity. This quickly becomes a problem for modern applications where it is desirable to limit the size of sensors. Adding a soft ferromangetic core with high relative permeability can largely increase the sensitivity of a coil, and hence allow for smaller sensor sizes^13^. A similar working principle has recently been used in the design of magnetic microwire coil-LC resonator sensors, but cobalt rich magnetic microwires are used instead of non-magnetic conductors such as copper^14^. The working principle of the sensor reported in this paper is fundamentally different than that of a conventional induction coil sensor. It relies on the changes in resonance frequency caused by external magnetic fields, instead of simply measuring the induction of the coil. It is also different from the magnetic microwire coil-LC resonator sensor^14^ that relies in changes in impedance of the microwire caused by external magnetic fields.

A simple model for a coil sensor is a lumped element representation of a non-ideal inductor. Winding cylindrical conductors close to each other will introduce parasitic elements ${R}_{par}$ and ${C}_{par}$, such that the non-ideal inductor can be represented as a series combination of an ideal inductor $L$ and ${R}_{par}$, in parallel with ${C}_{par}$^15–17^. The impedance of the coil ${Z}_{Coil}$ can then be written as

1 $${Z}_{{\rm{coil}}}=\frac{{R}_{par}+j\omega [{\rm{L}}(1-{\omega }^{2}{{\rm{LC}}}_{par})-{C}_{{\rm{par}}}{R}_{par}^{2}]}{{(1-{\omega }^{2}{\rm{L}}{C}_{par})}^{2}+{(\omega {C}_{par}{R}_{par})}^{2}},$$

where $\omega$ is the angular frequency, and $j$ is the imaginary unit. Resonance will occur when the inductive reactance $({X}_{L})$ and the capacitive reactance $({X}_{C})$ are equal in magnitude but differ in phase by 180 degrees. At this point very little current flows through the wire, the impedance ${Z}_{Coil}$ becomes very large and self-resonance is achieved^18,19^. The resonance frequency, ${f}_{0}$, is given by

2 $${f}_{0}=\frac{\sqrt{1-({{\rm{R}}}_{{\rm{par}}}^{2}{{\rm{C}}}_{{\rm{par}}}/{\rm{L}})}}{2\pi \sqrt{{{\rm{LC}}}_{{\rm{par}}}}}.$$

We expect a self-resonant behavior in the microwire coil as well, but the resonance frequency will be different than that of the inductor since the wire is now a magnetic material. We must also consider the effects of a ferromagnetic core on the sensor. The core will modify the relative permeability in the space within the coil, and this will in turn change the flux through the coil and hence affect the inductance. Since the permeability is field dependent, an external magnetic field will modify the permeability of the core and the resonance frequency with it. Additionally, we must consider that the wire itself is magnetic, which will lead to an effective permeability of the microwire and the core. The inductance of the sensor must then depend on this effective permeability, $L=L({\mu }_{eff}).$The sensitivity of the sensor is defined as the rate of change of the resonance frequency with respect to the external DC magnetic field,

3 $$Sensitivity=\frac{d{f}_{0}}{dH}.$$

The *Q* factor is also calculated by measuring the bandwidth, BW, and the resonance frequency using the following relation,

4 $$Q=\frac{{f}_{0}}{BW}$$

## Results and Discussion

**Figure 1:**
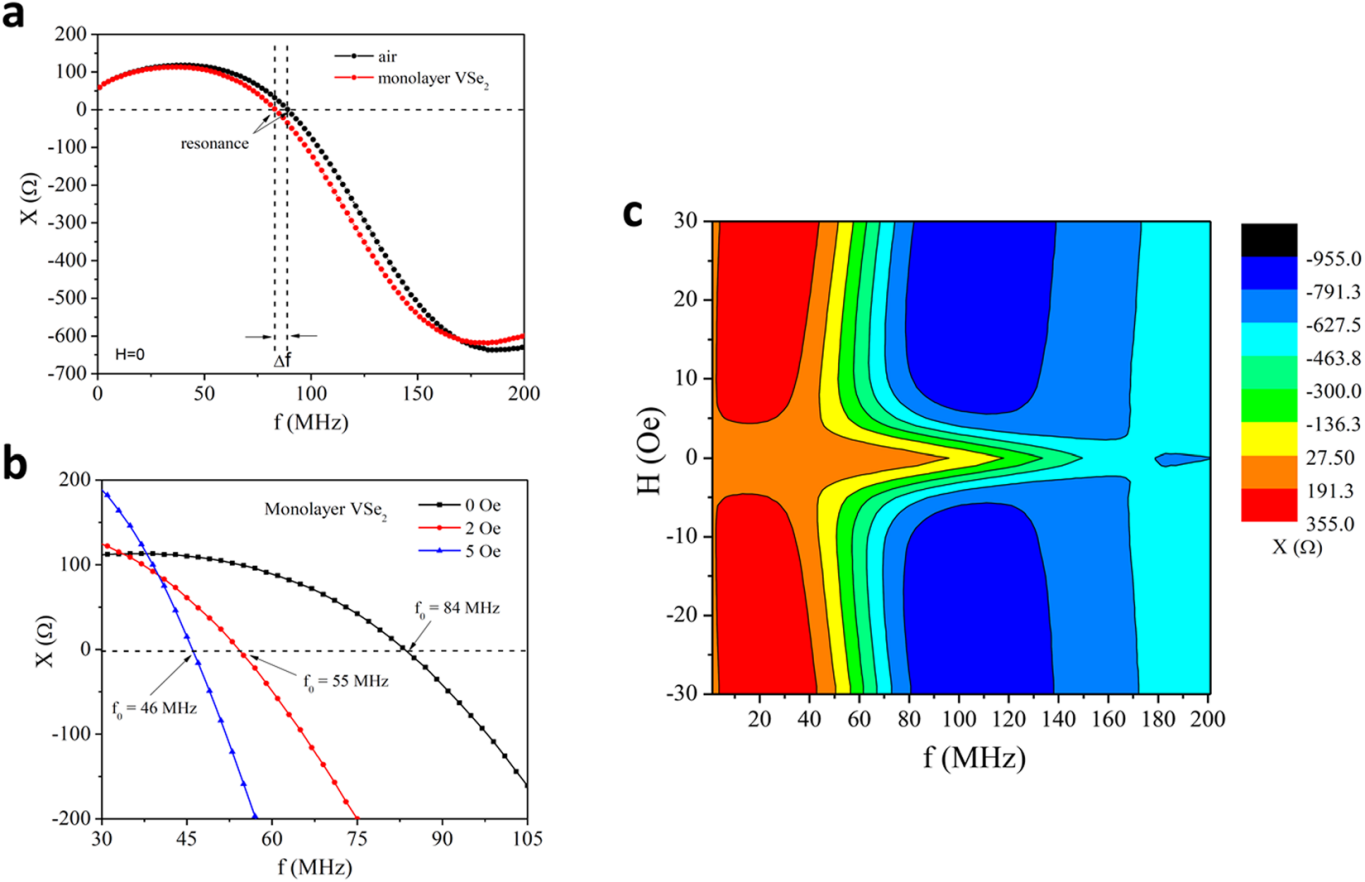
(**a**) Frequency dependence of the reactance of the sensor with and without the magnetic VSe_2_ film in the absence of an external magnetic field; (**b**) Frequency dependence of the reactance of the sensor with the magnetic VSe_2_ film shows large shifts in the resonant frequency with respect to applied magnetic fields; (**c**) 2D surface plot shows the magnetic field and frequency dependences of reactance.

A set of measurements is performed to characterize the sensor; the resonance frequency is found for every value of the field. Resonance is determined by the zero crossing of the reactance. To see the effects of the monolayer core we show in Fig. 1(a) the reactance of the coil with and without the monolayer core at zero field. The presence of the monolayer shifts the resonance frequency of the sensor by a few megahertz. We then applied the external field and observed how the reactance curve is shifted along with the corresponding resonance frequency as shown in Fig. 1(b), for the coil with the monolayer in its core. The reactance of the coil being proportional to the inductance changes drastically with frequency and applied field, as well as the resonance frequency (see Fig. (1c)).

**Figure 2:**
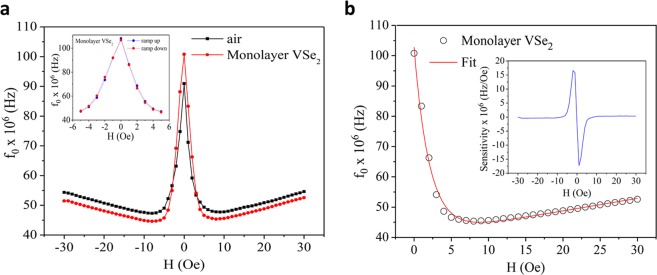
(**a**) The resonant frequency of the sensor (*f*_0_) changes as a function of magnetic field. A large change is achieved at fields between 0 and 5 Oe. A comparison between the air coil and the coil with the monolayer VSe_2_ core is shown. The inset shows that hysteresis in the sensor is negligible in the low field regime (below 5 Oe); (**b**) a fit of the magnetic field dependent resonant frequency *f*_0_(*H*) data is displayed, with the inset showing the magnetic field dependence of the sensor sensitivity.

The resonance frequency is determined for different values of an externally applied magnetic field, between −30 to 30 Oe. Figure 2(a) shows the change in resonance frequency as a function of applied field. Unique values of the resonance frequency exist for fields smaller than 5 Oe. A challenge we must consider when using a magnetic material is the hysteresis that is inherent to it. We have found that hysteresis in the sensor is negligible as long as fields no greater than 5 Oe are applied (see the inset of Fig. 2(a)). Since the sensor is intended to detect small magnetic fields, this should not hinder the sensor’s performance. At higher fields, the magnetic wire saturates, and ${f}_{0}$ changes very slowly, rending the sensor inoperable. A fit of the right branch of the ${f}_{0}$ plot is shown in Fig. 2(b) with the following equation,

5 $${f}_{0}=a+bH+c\ast {r}^{H},$$

where ${f}_{0}$ is the resonance frequency, $H$ is the external magnetic field and *a* = 40.50, *b* = 0.41, *c* = 62.30 and *r* = 0.62 are fitting parameters. When compared to the behavior of the air coil, the parameter *c* increases when the core is inserted; it is the parameter that depends the most on the properties of the core, hence modifying the contribution from the exponential term to the change in resonance frequency. While the wire dominates the behavior of the sensor, a magnetic core may be used to improve sensitivity. The sensitivity of the sensor is shown in the inset of Fig. 2(b). Values as large as 16 × 10^6^ Hz/Oe are achieved for the sensitivity. Future work must be focused on determining the minimum detectable signal of the sensor, but the high sensitivity may allow for applications such as bio-detection, where magnetic nanoparticles are used as bio-markers for pathogen detection and quantifying low particle concentrations is neccessary^20^. We also show how the VSe_2_ monolayer compares with a commercially available METGLAS 2714A ribbon (see Fig. S1). These ribbons have ultra-high DC permeability and are known to be excellent magnetic cores. The METGLAS ribbon shows a larger improvement in sensitivity than the single layer of VSe_2_. This is expected from a soft bulk material, but it shows that even a single layer of VSe_2_ can serve as a magnetic core to improve sensitivity of coil-based sensors, particularly in the low field regime.

**Figure 3:**
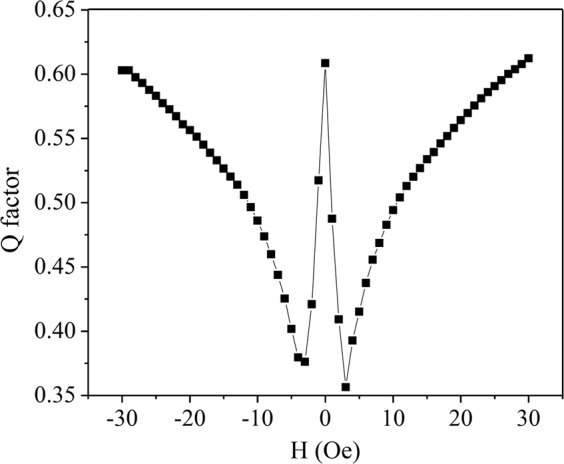
Magnetic field dependence of the Q factor of the sensor. The significant change in Q is observed at low field regime.

**Figure 4:**
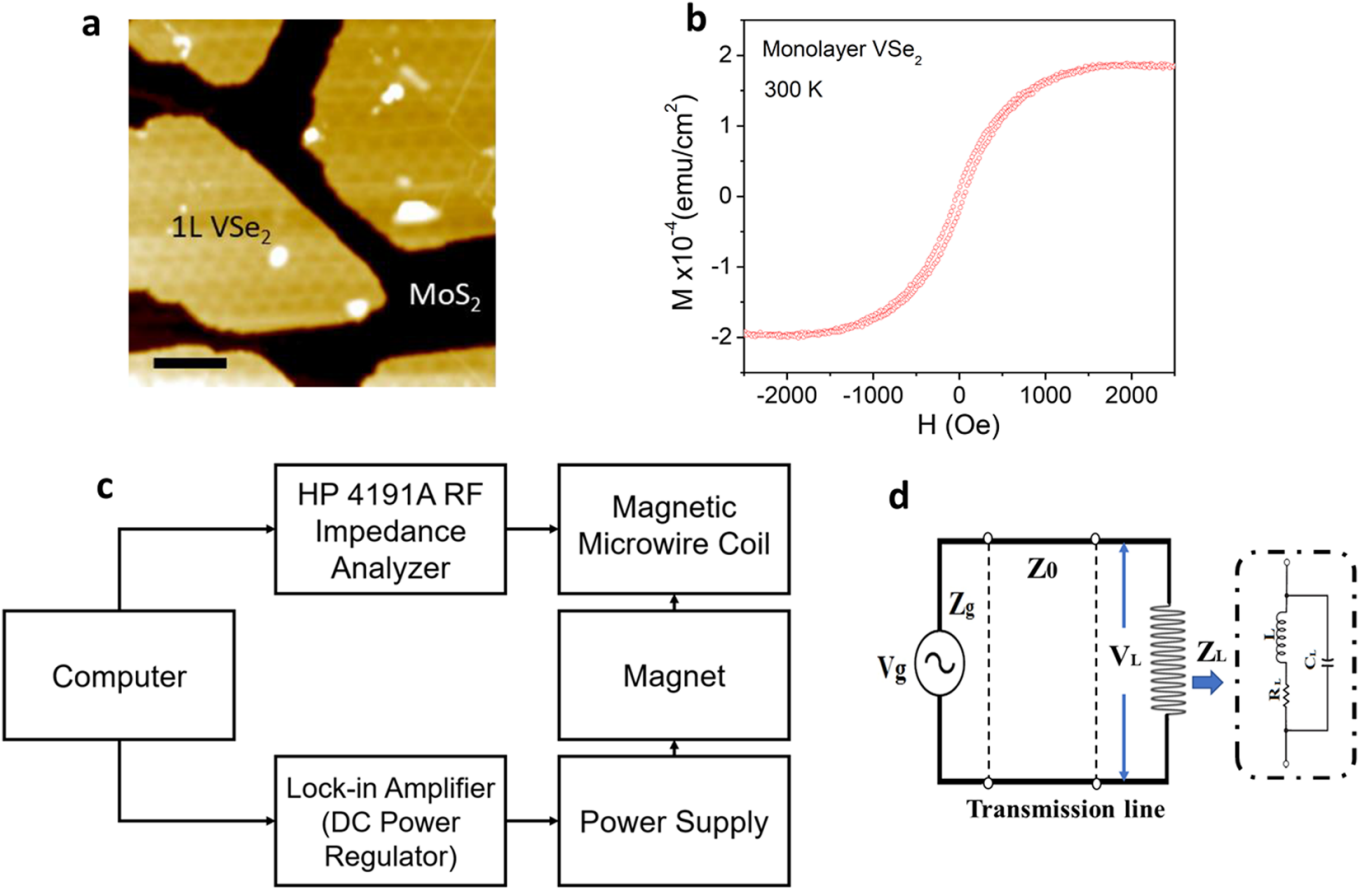
(**a**) A typical STM image of the single layer VSe_2_ film grown on single crystal MoS_2_; (**b**) the in-plane magnetic hysteresis (*M*-*H*) loop of the film measured at 300 K; (**c**) A block diagram of the measurement setup for testing performances of the sensor; (**d**) the LC-circuit composed of a magnetic microwire with the VSe_2_ film inserted in it.

Finally, we have determined the Q factor of the sensor at different values of external DC magnetic field, as shown in Fig. 3. We have found that for very small fields, smaller than 1 Oe, the Q factor is 0.6; as the field increases it reaches a minimum value of 0.36 at 3 Oe. Most of the losses are associated with the magnetic wire itself; the core slightly decreases the Q factor by less than 0.1 (see Fig. S2). This is consistent with the soft ferromagnetic characteristic of monolayer VSe_2_ (Fig. 4(b)), which predicts minimal magnetic losses.

In summary, a magnetic field sensor was built using a magnetic microwire coil as the sensing element with a single layer VSe_2_ film as the core. Sensitivity as large as 16 × 10^6^ Hz/Oe for very small fields was obtained. While conventional bulk materials with ultra-high permeability show a higher increment in the sensitivity of coil-based sensors, a single layered 2D magnet can still increase the sensor performance to a comparable level. Furthermore, core losses were shown to be minimal which is desirable for sensor design.

## Methods

To make the magnetic sensor, a Co_69.25_Fe_4.25_Si_13_B_12.5_Nb_1_ wire (diameter, ~60 μm) was wound into a 15-turn, 10 mm long coil with a 5 mm internal diameter. The fabrication details and material characterization of the microwire can be found elsewhere^21,22^. Monolayer VSe_2_ was used as the core of the coil. The single layer films of VSe_2_ were grown on graphite (HOPG) and single crystal MoS_2_ by molecular beam epitaxy (MBE); the details of which have been reported in our previous work^3^. Since both monolayer VSe_2_ samples grown on MoS_2_ and HOPG show similar magnetic and sensing properties at ambient temperature, in this paper we only report on the properties of monolayer VSe_2_ grown on MoS_2_, but the sensor characterization data for monolayer VSe_2_ grown on HOPG can be found in Fig. S1. We note that HOPG is diamagnetic in nature and has no appreciate change in response to magnetic field^3^. Figure 4(a) shows a typical scanning tunnel microscopy (STM) image of the VSe_2_ film. It can be seen that the single layer of VSe_2_ was epitaxially grown on the MoS_2_ substrate. The magnetization versus magnetic field (*M*-*H*) curves, measured by a vibrating sample magnetometer (VSM) on VSe_2_ grown on both HOPG and MoS_2_, show a soft ferromagnetic characteristic (small coercive field, small remanent magnetization, and high saturation magnetization) of the single layer VSe_2_ film at room temperature (Fig. 4(b)), which is desirable for its use as the core of the sensor that itself operates at room temperature. Sensor characterization and magnetic measurements were performed by applying an in-plane magnetic field, along the easy axis of the monolayer VSe_2_. The core was ~14 mm long and ~4 mm wide, allowing us to insert and remove the core with ease. The sensor was mounted on a test fixture made of a dielectric material on top of a copper ground plane; SMA connector ports were soldered to the ground plane on two ends and the two leads of the inductor were soldered to the center pin of each connector.

One port reflection measurements were performed with an HP 4191A impedance analyzer over the frequency range 1–200 MHz. A simple short-open-load (SOL) calibration was made. The test fixture was connected to the impedance analyzer through a coaxial cable, and it was terminated with a 50 ohm cap. The effects of the cable were removed by the calibration. The impedance (*Z*), resistance (*R*), and reactance (*X*) are calculated from the reflection measurement. An external DC magnetic field was generated by a Helmholtz coil. A diagram of the setup is shown in Fig. 4(c,d). The external field was applied transverse to the coil axis and in plane of the 2D magnet. A frequency sweep was performed for every value of the field which was swept from −30 to 30 Oe in steps of 1 Oe.

## Supplementary information

Supplementary Information.

## Data Availability

The datasets generated during and/or analyzed during the current study are available from the corresponding author on reasonable request.
