# Supplementary material for: A magnetic sensor using a 2D van der Waals ferromagnetic material
Source: Sci Rep. 2020 Mar 16;10:4789. doi: 10.1038/s41598-020-61798-2 (PMC7075862; doi:10.1038/s41598-020-61798-2)
Supplement: Supplementary file 1 — Supplementary Information. [file 41598_2020_61798_MOESM1_ESM.docx]

**Supplementary Information**

**A magnetic sensor using a 2D van der Waals ferromagnetic material**

Valery Ortiz Jimenez^1^, Vijaysankar Kalappattil^1^, Tatiana Eggers^1^, Manuel Bonilla^1^, Sadhu Kolekar^1^, Pham Thanh Huy^2^, Matthias Batzill^1^, and Manh-Huong Phan^1,*^

^1^ Department of Physics, University of South Florida, Tampa, FL 33620, USA

^2^ Phenikaa Institute for Advanced Study, Phenikaa University, Yen Nghia, Ha-Dong District, Hanoi 1000, Viet Nam

Email: [phanm@usf.edu](mailto:phanm@usf.edu)

**1. Comparison of sensor performance using a conventional METGLAS 2714A ribbon core and a single layer of VSe_2_.**

**
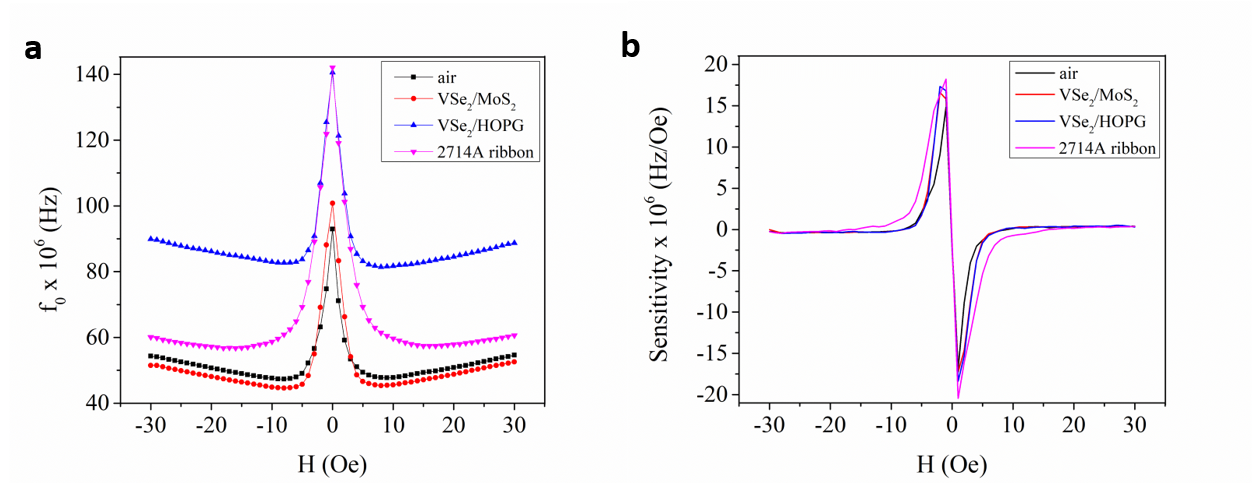
**

**Fig. S1:** (a) The resonant frequency of the sensor (*f*_0_) changes as a function of magnetic field, for the air coil, the coil with a monolayer VSe_2_ core on single crystal MoS_2_ and HOPG, and the coil with a conventional METGLAS 2714A ribbon core; (b) Magnetic field dependence of the sensor sensitivity when using the air coil, the coil with a monolayer VSe_2_ core on single crystal MoS_2_ and HOPG, and the coil with a conventional METGLAS 2714A ribbon core respectively.

**2. Q factor of the sensor using the air coil.**

**
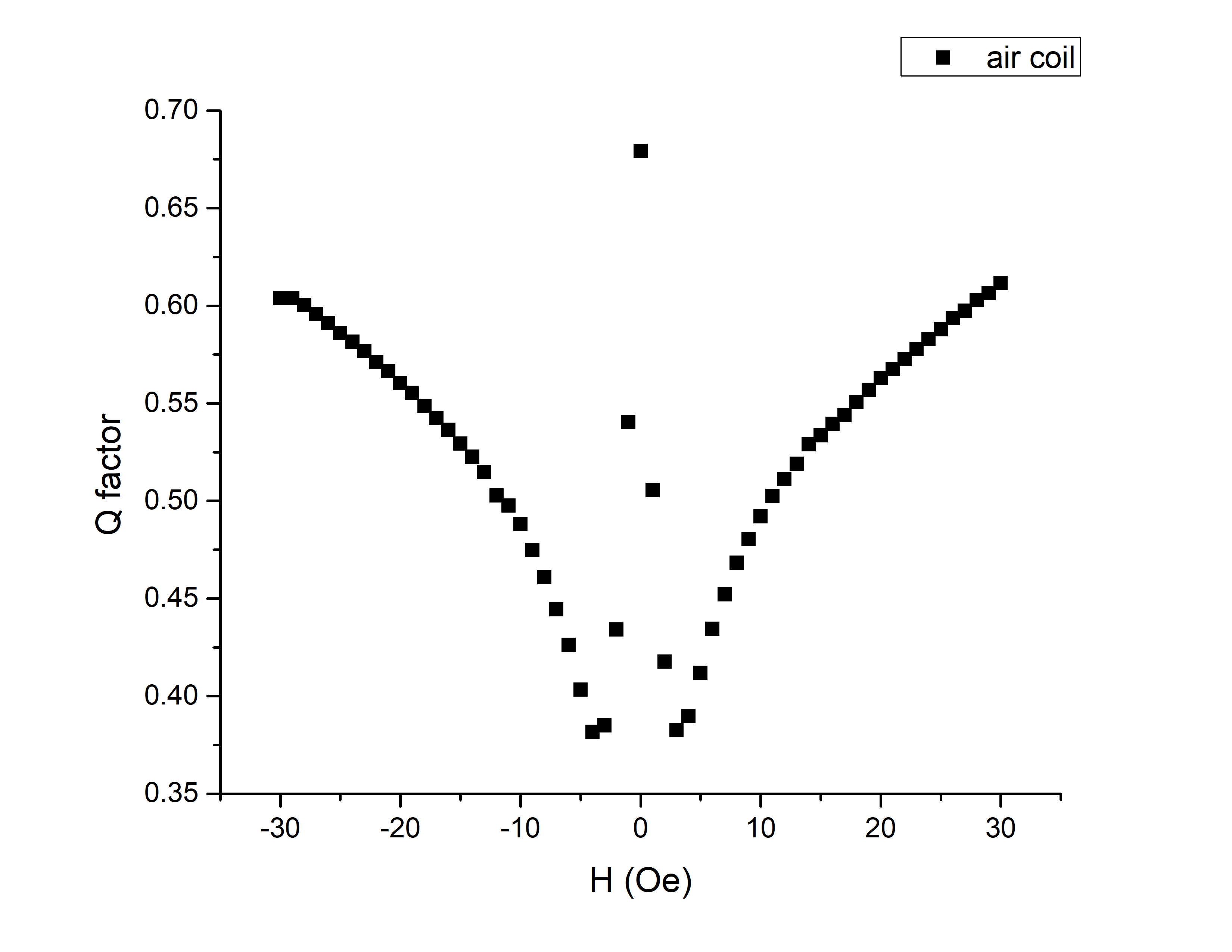
**

**Fig. S2:** Magnetic field dependence of the Q factor of the sensor using the air coil only.
